# Early decrease in blood lymphocyte count is associated with poor prognosis in COVID-19 patients: a retrospective cohort study

**DOI:** 10.1186/s12890-023-02767-z

**Published:** 2023-11-20

**Authors:** Gong Chen, Xiaofang Zhao, Xinglin Chen, Chengyun Liu

**Affiliations:** 1 Department of Anesthesiology, the Third Xiangya Hospital, Central South University, Changsha, China; 2 Department of Geriatrics, Union Hospital, Tongji Medical College, Huazhong University of Science and Technology, Wuhan, China; 3 Department of Epidemiology and Biostatistics, Empower U, X&Y Solutions Inc., Boston, MA USA; 4 The First People’s Hospital of Jiangxia District, Wuhan City & Union Jiangnan Hospital, Huazhong University of Science and Technology, Wuhan, China

**Keywords:** Lymphocyte count, COVID-19, Hospital mortality

## Abstract

**Background:**

Previous studies have declared that baseline lymphocyte count is associated with COVID-19-related death. However, whether dynamic lymphocyte change over time affects prognosis in COVID-19 patients is unknown. This study aims to investigate the significance of lymphocyte count during the progression of the disease in COVID-19 patients.

**Methods:**

The retrospective cohort study recruited COVID-19 patients at the First People’s Hospital of Jiangxia District in Wuhan from January 7, 2020, to February 28, 2020. The demographics, medical histories, results of the blood routine test, and patients’ outcomes were collected. We utilized a generalized additive mixed model to compare trends in lymphocyte count over time among survivors and non-survivors, with an adjustment for potential confounders. The statistical analysis used R software and EmpowerStats. Significance was determined at a *P*-value of less than 0.05 (two-sided).

**Results:**

A total of 532 patients were included in the study. Overall, there were 29/532 in-hospital deaths (5.45%). Lymphocytes declined over time in the non-survivor group and increased in the survivor group in the first 10 days of hospitalization. Within 10 days after admission, lymphocyte count increased in the survivor group and decreased in the non-survivor group. The difference in lymphocyte counts between survivors and non-survivors increased by an average of 0.0732 × 10^9^/L daily. After adjusting for several covariables, the increasing value remained at 0.0731 × 10^9^/L per day.

**Conclusion:**

In the early stage, lymphocyte count can dynamically reflect the pathophysiological changes in COVID-19 patients. An early decrease in lymphocyte count is associated with mortality in COVID-19 patients.

## Background

The coronavirus disease 2019 (COVID-19) pandemic is the most severe infectious disease outbreak this century, causing millions of infections and deaths worldwide. While the pandemic has primarily abated in many regions, seasonal epidemics and periodic pandemics remain a constant threat and cause significant mortality [1, 2]. Until today, COVID-19 infection can cause many complications [2, 3], some may deteriorate and even die, especially in high-risk patients [3–6]. Therefore, early identification of high-risk patients is crucial to ensure prompt and appropriate treatment for COVID-19 patients.

Dysregulation of the immune system is directly linked to disease severity in COVID-19 patients [7]. Immunocompromised individuals are at increased risk for COVID-19-associated mortality due to immunologic deficits that limit the virus’s clearance [7]. Zhao et al. reported that neutrophilia, lymphocytopenia, low CD4^+^ T cells, and decreased C3 could predict the mortality of COVID-19 patients [8]. A meta-analysis reported that lymphocyte count reduction correlated with illness severity in COVID-19 patients [9]. Although several studies have investigated the relationship between lymphocyte count and severity in COVID-19 patients, there is still limited information on the dynamic changes in lymphocyte count over time in COVID-19 patients, which is more important for determining patients’ conditions. In the present study, we aim to investigate the trends of lymphocyte count during the progression of the disease in COVID-19 patients.

## Methods

### Study design and participants

This study recruited 1066 patients diagnosed with COVID-19 at the First People’s Hospital of Jiangxia District in Wuhan between January 7, 2020, and February 28, 2020. Patients who meet these criteria would be excluded: (1) those still hospitalized by February 29, 2020 (345 cases); (2) those who died on admission (5 cases); (3) those with malignancy (6 cases); (4) those with a history of gastrointestinal surgery (7 cases); (5) those baseline lymphocyte counts were missing (31 cases); (6) those lymphocyte counts detected only once (143 cases). Finally, 532 patients were included. This study was approved by the Medical Ethics Committee of the First People’s Hospital of Jiangxia District in Wuhan. Written informed consent was not required because the data were anonymous, and the study was retrospective observational.

### Measurement of the covariates

The demographics, medical histories, and outcomes were collected from the electronic hospital information system. Blood samples were collected in the morning after an overnight fast, and blood routine tests were conducted. We got white blood cell count, neutrophil count, lymphocyte count, and platelet count from the hospital information system. Lymphocyte count was rechecked at variable intervals.

### Statistical analysis

**Fig. 1:**
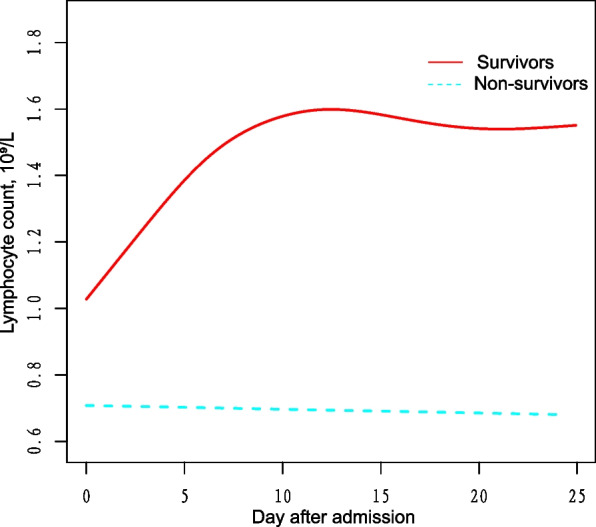
Association between dynamic change in lymphocyte count over time and in-hospital mortality. A non-linear relationship was found between changes in lymphocyte count over time and in-hospital mortality by GAMM. The smooth curve fitting graph shows the changes in lymphocyte counts of both survivors and non-survivors over time. The adjusted covariates include age, sex, baseline white blood cell count, neutrophil count, platelet count, history of hypertension, and history of diabetes

Categorical data are displayed in absolute counts (percentages), while measured data are presented as mean ± standard deviation. In the baseline characteristics of survivors and non-survivors (Table 1), sex, hypertension, and diabetes were compared with chi-square test, and age, leukocytes, neutrophils, lymphocytes, and platelets, which followed a normal distribution, were compared with ANOVA. The changes in lymphocyte count over time between groups (Fig. 1) and the relationship between the early change of lymphocyte count and death in COVID-19 patients (Table 3) were evaluated using a generalized additive mixed model (GAMM). GAMM proves to be highly effective in analyzing repeated measurement outcomes, particularly when some data are missing, there are inconsistent intervals between measurements, and moderate sample size [10, 11]. The statistical analysis used R software (http://www.R-project.org, The R Foundation) and EmpowerStats (http://www.empowerstats.com, X&Y Solutions, Inc., Boston, MA). Significance was determined at a *P*-value of less than 0.05 (two-sided).

**Table 1:** Baseline characteristics

Characteristics	Survivors, *n* = 503	Non-survivors, *n* = 29	*P*-value
Age, years	48.18 ± 14.41	64.72 ± 13.11	< 0.001
Sex			0.821
Male	232 (46.12%)	14 (48.28%)	
Female	271 (53.88%)	15 (51.72%)	
White blood cells, 10^9^/L	5.43 ± 3.13	7.60 ± 3.28	< 0.001
Neutrophils, 10^9^/L	3.84 ± 2.89	6.31 ± 3.32	< 0.001
Lymphocytes, 10^9^/L	1.08 ± 0.49	0.76 ± 0.36	< 0.001
Platelets, 10^9^/L	186.09 ± 72.35	176.14 ± 89.00	0.478
Hypertension			< 0.001
No	411 (81.71%)	13 (44.83%)	
Yes	92 (18.29%)	16 (55.17%)	
Diabetes			< 0.001
No	453 (90.06%)	20 (68.97%)	
Yes	50 (9.94%)	9 (31.03%)

## Results

### Characteristics of patients

The demographic and clinical characteristics at baseline are shown in Table 1. The overall in-hospital mortality rate was 5.45% (29/532). Especially, non-survivors were significantly older than survivors (64.72 ± 13.11 vs. 48.18 ± 14.41, *P* < 0.001). Compared with the survivors, the counts of white blood cells (7.60 ± 3.28 vs. 5.43 ± 3.13, *P* < 0.001) and neutrophils (6.31 ± 3.32 vs. 3.84 ± 2.89, *P* < 0.001) were higher in non-survivors. In addition, the counts of baseline lymphocytes (0.76 ± 0.36 vs. 1.08 ± 0.49, *P* < 0.001) and platelets (176.14 ± 89.00 vs. 186.09 ± 72.35, *P* < 0.001) were lower in non-survivors than in survivors. Regarding comorbidities, it was observed that non-survivors were more likely to have a history of hypertension (*P* < 0.001) and diabetes (*P* < 0.001). The differences in lymphocyte count between survivors and non-survivors at different time points are presented in Table 2. On the 5-6th day, male survival patients had a significantly higher lymphocyte count than non-survivors (*P* < 0.001). Similarly, female survival patients had a significantly higher lymphocyte count on the 14-15th day than non-survivors (*P* = 0.023).

**Table 2:** The difference in lymphocyte count between survivors and non-survivors stratified by sex

Time	Lymphocyte count, 10^9^/L, Mean (SD) median (IQR)		
Male			
	**Survivors,** ***n*** **= 232**	**Non-survivors,** ***n*** **= 14**	***P*-value**
On admission	1.06 (0.52) 0.95 (0.69, 1.31)	0.77 (0.37) 0.76 (0.58, 0.92)	0.026
On 5-6th day	1.40 (0.77) 1.40 (0.71, 1.99)	0.55 (0.32) 0.49 (0.35, 0.61)	< 0.001
On 14-15th day	1.45 (0.85) 1.39 (0.96, 1.63)	0.60 (0.25) 0.60 (0.51, 0.69)	0.080
Female			
	**Survivors,** ***n*** **= 271**	**Non-survivors,** ***n*** **= 15**	***P*-value**
On admission	1.09 (0.47) 1.03 (0.74, 1.38)	0.75 (0.37) 0.65 (0.54, 0.99)	0.004
On 5-6th day	1.48 (0.78) 1.30 (0.89, 1.97)	1.05 (0.52) 1.05 (0.87, 1.24)	0.4
On 14-15th day	1.58 (0.65) 1.50 (1.02, 2.09)	0.72 (0.59) 0.59 (0.32, 0.99)	0.023

### The relationship between change in lymphocyte count over time and in-hospital mortality

Figure 1 illustrates the variations in lymphocyte count over time for survivors and non-survivors. Briefly, lymphocytes declined over time in the non-survivor group. However, in the survivor group, lymphocytes increased in the first 10 days and maintained stably in the following days. Furthermore, we compared survivors and non-survivors to understand better the correlation between the initial alteration (0–10 days) in lymphocyte count and the in-hospital mortality rate. Table 3 illustrates a significant increase in the lymphocyte count difference between the survivor and non-survivor groups within 10 days of admission. On average, this difference increases by 0.0732 × 10^9^/L daily. After adjusting for several covariables (sex, age, white blood cell count, neutrophil count, platelet count, history of hypertension, and history of diabetes), the increasing value remained at 0.0731 × 10^9^/L per day, indicating the result was stable.

**Table 3:** Relationship between early (0–10 days) changes in lymphocyte count (10^9^/L) and death in COVID-19 patients (from GAMM)

Outcome	Model I		Model II	
	β (95%CI)	*P*-value	β (95%CI)	*P*-value
Intercept	1.5131 (1.3451, 1.6811)	< 0.0001	0.4872 (0.3185, 0.6559)	< 0.0001
Day	0.0633 (0.0553, 0.0714)	< 0.0001	0.0632 (0.0553, 0.0710)	< 0.0001
Death	−0.1073 (−0.3330, 0.1183)	0.3516	0.1077 (−0.0733, 0.2886)	0.2440
Day×Death	−0.0732 (− 0.1040, − 0.0424)	< 0.0001	−0.0731 (− 0.1028, − 0.0434)	< 0.0001

β: the effect value of lymphocyte count over time; CI: confidence interval; Intercept: lymphocyte count at day = 0 and death = 0 (lymphocyte count at admission in the survival group); Day: the increasing value of lymphocyte count at death = 0 over time (changes in survival group lymphocyte count with length of hospital stay); Death: the difference of lymphocyte count at day = 0 between the group of death = 1 and the group of death = 0 (differences in lymphocyte counts between the surviving and non-surviving groups at admission); Day × death, the average increase in lymphocyte count daily under the condition of the group of death = 1 compared with the group of death = 0 (average difference in lymphocyte count changes between the surviving and non-surviving groups on a daily basis); Model I: adjusted for sex and age; Model II: adjusted for sex, age, white blood cell count, neutrophil count, lymphocyte count, history of hypertension, history of diabetes

## Discussion

This study investigated the trends of lymphocyte count during the progression of the disease in COVID-19 patients. It was found that lymphocytes declined over time in non-survivors and increased in survivors in the early hospitalization stage. Within 10 days after admission, the difference in lymphocyte count between the two groups increases by 0.0731 × 10^9^/L per day.

Many studies have explored the correlation between lymphocytes and COVID-19 hospitalization outcomes. Xiong et al. have indicated that COVID-19 patients, particularly in severe cases, tend to have a lower count of lymphocytes [12]. Furthermore, Niu et al. suggested that decreased lymphocyte count in hospitalized COVID-19 patients is independently associated with increased risk of in-hospital mortality [13]. Some researchers have even found that the ratio of neutrophil count to lymphocyte count (NLR) can serve as a predictive factor for COVID-19 hospital death [14, 15]. However, these studies only focused on the correlation between baseline lymphocyte count (lymphocyte count at admission) and hospitalization outcomes. Yet, clinicians are more concerned about changes in lymphocyte count after hospitalization, as changes in test results may indicate whether the patient has improved after treatment. Our studies have observed the trends in lymphocyte counts over time in survivors and non-survivors of COVID-19, which could reflect the changes in patients’ conditions. Therefore, compared to previous studies on the correlation between baseline lymphocyte count and prognosis in COVID-19 patients, our study can better reflect the survival probability of these patients after treatment. To our knowledge, this study is the first to examine the changes in lymphocyte counts over time among survival and non-survival patients with COVID-19.

### Strengths and limitations

Our study has several strengths. First, the present study is more practical than the previous studies that only examined baseline lymphocyte count. Second, as the lymphocyte count was measured repeatedly in this study, using GAMM was appropriate in elucidating the correlation between the fluctuation of lymphocyte count and the clinical outcomes. Third, we used strict statistical adjustments to reduce potential confounding, and the result remains stable after adjusting for several covariables.

Our research has some limitations. First, the study only included Chinese COVID-19 patients, so it may not be generalized to people from other countries. Second, this is an observational cohort study, so causal conclusions cannot be drawn. Further study is needed to investigate whether improving immunity to increase lymphocyte count can improve the prognosis of COVID-19 patients. Third, the sample size of this study was relatively small. However, the sample size is sufficient to get a conclusion. Future studies with larger sample sizes are needed to determine the association of changes in lymphocyte count over time and in-hospital mortality in COVID-19 patients.

## Conclusion

In the early stage, lymphocyte count can dynamically reflect the pathophysiological changes in COVID-19 patients. An early decrease in lymphocyte count is associated with mortality in COVID-19 patients. This finding may indicate that COVID-19 patients with decreased lymphocytes after admission need more attention.

## Data Availability

The datasets used and/or analyzed during the current study are available from the corresponding author on reasonable request.

## Abbreviations

COVID-19: Coronavirus Disease 2019
ANOVA: Analysis of variance
GAMM: Generalized additive mixed model
